# Extending the Shelf-Life of Fresh-Cut Green Bean Pods by Ethanol, Ascorbic Acid, and Essential Oils

**DOI:** 10.3390/foods10051103

**Published:** 2021-05-17

**Authors:** Asmaa H. R. Awad, Aditya Parmar, Marwa R. Ali, Mohamed M. El-Mogy, Karima F. Abdelgawad

**Affiliations:** 1 Vegetable Crops Department, Faculty of Agriculture, Cairo University, 12613 Giza, Egypt; soma_h_97@yahoo.com (A.H.R.A.); karima.abdelgawad@agr.cu.edu.eg (K.F.A.); 2 Natural Resources Institute, University of Greenwich, Central Avenue, Chatham Maritime, Kent ME4 4TB, UK; 3 Food Science Department, Faculty of Agriculture, Cairo University, 12613 Giza, Egypt; marwa3mrf@agr.cu.edu.eg

**Keywords:** *Phaseolus vulgaris*, peppermint, tea tree, storability, minimal processed, ready to eat

## Abstract

Green beans are a perishable crop, which deteriorate rapidly after harvest, particularly when minimally processed into ready-to-eat fresh-cut green beans. This study investigated the effectiveness of ethanol, ascorbic acid (AsA), tea tree essential oil (TTO), and peppermint essential oil (PMO) on the quality and storability of fresh-cut green bean pods samples stored at 5 °C for 15 days. Our results indicated that samples treated with ethanol, AsA, TTO, and PMO preserved appearance, firmness (except ethanol), chlorophyll content, and moisture compared with the samples without any treatment (control). Additionally, higher vitamin C, total soluble solids (TSS), total sugars, and total phenolic compounds (TPC) were observed in samples treated with ethanol, AsA, TTO, and PMO compared with the control. The most effective treatments for controlling microbial growth were ethanol followed by either TTO or PMO. All the treatments had positive effects on shelf life, maintained quality, and reducing microbial growth during 15 days of cold storage. A particular treatment can be selected based on the economic feasibility and critical control point in the value chain.

## 1. Introduction

Green bean (*Phaseolus vulgaris*) belongs to the family of *Fabaceae* and is considered one of the most important legume crops worldwide. The pods of green bean can be harvested at an immature stage (as a fresh vegetable) or mature stage (for dried seeds). Green bean is a rich source of minerals, vitamins, and dietary fibre that play a significant role in the human diet and wellbeing [1]. However, green bean pods are highly perishable with limited shelf-life due to their high respiration rate. During postharvest, green beans are susceptible to mechanical damage, shriveling, chlorophyll pigment degradation, and increased fibre content [2,3]. These biochemical changes reduce the quality and consumption of green bean pods and decrease their economic and nutritional values.

Due to rapid urbanisation in developing countries, demand for fresh minimally processed refrigerated fruit and vegetable has increased significantly. Minimal processing includes trimming, peeling, coring, cutting, and packing. These unit operations result in some undesirable morphological and physiological changes such as browning, pigmentations, and microbial growth. Additionally, the moisture loss and respiration rate of minimally processed vegetables are much higher during refrigerated storage compared to non-processed vegetables.

The effects of several bioactive compounds in essential oils (EOs) and plant extracts as anti-microbial and shelf life enhancing agents in horticultural crops are well known [4,5]. The United States Food and Drug Administration (FDA) classified many EOs as safe, nontoxic, antioxidant, and anticancer compounds including tea-tree essential oil (TTO) and peppermint essential oil (PMO) [6,7]. TTO and PMO are obtained from *Melaleuca alternifolia* and *Mentha piperita* by hydrodistillation, respectively. Several previous studies have reported the positive effect of TTO for controlling postharvest diseases of fresh fruit and vegetables such as strawberry [8] and lettuce [9]. It has been reported that calcium chloride + chitosan + TTO emulsion treatment preserved the firmness and overall quality of fresh-cut red bell pepper for 18 d at 4 °C [10]. New investigations on the effect of PMO as a postharvest treatment showed preserved quality and storability of fresh fruit such as table grapes [11] and dragon fruit [12].

Ascorbic acid (AsA) plays an important role in plant antioxidant systems and human health [13,14]. Positive effects of AsA for controlling enzymatic browning in fruit and vegetables such as plums [15], mung bean sprouts [16], and fresh-cut artichoke [17] has been reported previously. Ethanol is another natural compound that is used in various postharvest treatments. Ethanol is considered safe as per the Generally Recognized As Safe (GRAS) guidelines [18]. Previous reports mentioned that postharvest ethanol treatments (dips or vapour) extend the storage duration of several fresh horticultural products. For example, ethanol has been shown to reduce postharvest fungal diseases of table grapes [19] and Chinese berries [20], delay yellowing of broccoli florets [21], retard senescence in vegetables [22], inhibit the ethylene pathway biosynthesis of melons [23], and suppress the ripening of tomatoes [24].

To the best of the author’s knowledge, this is a novel investigation. There were no previous reports on the effect of ethanol, AsA, TTO, and PMO on postharvest behaviour and quality of green beans. The objective of the current study was to assess the effect of these natural compounds on the quality parameters and shelf life of fresh-cut green beans stored for 15 d at 5 °C.

## 2. Materials and Methods

### 2.1. Preparation of Plant Material and Treatments

Green bean pods (*Phaseolus vulgaris* L., variety Hama) were harvested from a local private farm and transferred under cooling condition (4 °C) within two hours to the postharvest laboratory. Green bean pods free from defects and damage, with uniform diameter and length, were prepared by cutting the two ends of the pod with a sterile sharp knife. The fresh-cut green bean pods were immersed in four different treatment solutions:

A. Ethanol (0.5%),
B. Ascorbic acid (AsA) (0.5%),
C. Tea tree oil (TTO) (0.5%), and
D. Peppermint oil (PMO) (0.5%).

The control group left without any treatment. TTO and PMO concentrations were prepared by dissolving the required amounts of oils in 50 mL of 0.05 mL Tween-20 and then sterile distilled water was added to obtain 1000 mL of desire concentrations. The treated samples were then dried in a laminar airflow hood for 2 h, and then packed and sealed in micro-perforated (five perforations/cm^2^, perforation diameter 0.7 mm) polypropylene bags by using autoclaved forceps. The dimensions of the bags were as follows: length 22 cm, breadth 16.5 cm, and 1 mm thickness; the bags contained 250 g of samples each. Samples were stored at 5 °C and 90% RH for 15 d. Each treatment was carried out in triplicate and the whole experiment was repeated. For each treatment, samples were divided into two groups. One group was used to determine weight loss, decay, and general appearance throughout full storage time and the other was used to determine pod quality parameters (firmness and TSS), chemical compounds (vitamin C, TPC, and chlorophyll content), mould, yeast, and total microbial count. All parameters were measured at time intervals of 0, 3, 6, 9, 12, 15 d after treatments.

### 2.2. Gas Chromatography-Mass Spectrometry Analysis (GC-MS)

To analyse the chemical compositions of the TTO and PMO, the GC-MS system (Agilent Technologies) equipped with gas chromatography (7890B) and mass spectrometer detector (5977A) (Shimadzu, Kyoto, Japan) was used. Samples were diluted with hexane (1:19, *v*/*v*). The GC was equipped with an HP-5MS column (30 m × 0.25 mm internal diameter and 0.25 μm film thickness). Analyses were carried out using helium as the carrier gas at a flow rate of 1.0 mL/min at a split ratio of 1:10, injection volume of 1 µL and the following temperature program: 40 °C for 1 min; rising at 4 °C min^−1^ to 150 °C and held for 6 min; rising at 4 °C min^−1^ to 210 °C and held for 5 min. The injector and detector were held at 280 °C and 220 °C, respectively. Mass spectra were obtained by electron ionization (EI) at 70 eV; using a spectral range of m/z 40 to 550 and solvent delay of 3 min. The identification of different constituents was determined by comparing the spectrum fragmentation pattern with those stored in Wiley and NIST Mass Spectral Library data.

### 2.3. Free Radical Scavenging Using DPPH

The free radical scavenging activity of the TTO and PMO against 2,2-diphenyl-1-picrylhydrazyl (DPPH) was measured according to the method described in [25]. First, EOs samples were diluted with methanol (50 µL mL^−1^); then, the sample was mixed with 4 mL DPPH solution (0.24 mg L^−1^ in methanol). The control sample was prepared by mixing methanol with DPPH solution at the same volume. The mixture was mixed well and incubated in the dark at room temperature for 30 min; then, the absorbance was measured at 517 nm using a spectrophotometer (Unico UV-2000, UNICO company, Fairfield, NJ, USA). The free radical scavenging activity of each essential oil was calculated according to Equation (1).

*Free radical scavenging (%) = [(A _control_ − A _sample_)/A _control_]* × 100 (1)

where: *A _control_ = absorbance of control sample*, and *A _sample_ = absorbance of essential oil sample*.

### 2.4. Determination of Weight Loss, General Appearance, Firmness, and Total Soluble Solids

Green bean samples were weighed immediately after (drying in a laminar airflow hood for 2 h) and at every sampling time to measure weight loss by using a digital laboratory scale. Another set of the green bean samples of 250 g each in triplicates) were used for further chemical analysis. To determine general appearance, the following scale was used: 9 = excellent, 7 = very good, 5 = good, 3 = poor, and 1 = unacceptable. The appearance score was assessed by a group of three trained laboratory panelists.

The firmness values of each pod were determined at three different points by using the digital penetrometer (PCE-PTR 200, PCE Americas Inc., Jupiter, FL, USA) with 6 mm diameter probe (range 0 to 1 kg*) [26]. The firmness values were expressed in Newton (N). To determine total soluble solids (TSS), a digital refractometer (model PR101, Atago [0–45%] Co. Ltd., Tokyo, Japan) was used at room temperature [27].

### 2.5. Determination of Total Sugar, Vitamin C, Total Phenolic Compounds, and Chlorophyll Content

The total sugar content was determined by the anthrone method at 630 nm as described in [28]. Briefly, 200 mg fruit were extracted three times with ethanol (80%) at 80 °C. Then, the extracts were evaporated to dryness and redissolved in 2 mL distilled water. One millilitre of sample extracts was added to 1.5 mL of anthrone reagent (0.2% in H_2_SO_4_) and mixed thoroughly. The sample was brought to boil using a boiling water bath. The solution was cooled to room temperature and absorbance was measured. The formation of the blue-green complex indicates the presence of total sugars. Glucose was used as a standard. Vitamin C content was determined using the titrimetric method with 2,6-dichlorophenolindophenol described by the Association of Official Analytical Chemistry [29].

TPC was calculated by using the Folin–Ciocalteu reagent with some alteration by using gallic acid as a standard curve [17]. Five grams of the sample was diluted using 5 mL of methanol (80%). The solution was blended with 2.5 mL of Folin–Ciocalteu (10-fold with distilled water) and added to 2.5 mL of distilled water. Afterwards, 2 mL of aqueous sodium carbonate solution (7.5%, *w*/*v*) was added after incubation for 5 min. The final solution was mixed and incubated in the dark at room temperature for 1 h. The absorption was assessed at 765 nm using the spectrophotometer, and the results were expressed as milligrams of gallic acid equivalent (GAE) per 100 mg of fresh fruit weight.

Chlorophyll content was determined as described in [30]. In brief, 0.5 g of fresh sample were homogenized with 5 mL dimethyl formamide and kept in the dark in the refrigerator for 48 h. The absorbance was then measured at 470, 647 and 663 nm with a spectrophotometer (model UV-2401 PC, International Equipment Trading LTD. (IET), Milano, Italia).

### 2.6. Determination of Mould, Yeast and Total Microbial Count

Ten grams of each treatment were homogenized with 90 mL sterile saline for 2 min by a stomacher (Stomacher BW-400P, Turelab, Shanghai, China). Total count and mould and yeast count (MY) were enumerated on plate count agar and potato dextrose agar after incubation at 37 °C for 48 h and 28 °C for 5 d, respectively [31]. The results were expressed as log10 of colony-forming units per gram sample (CFU g^−1^).

### 2.7. Statistical Analysis

Statistical analyses of the pooled data from the two experiments were performed with SPSS software. The data were analyzed by one-way analysis of variance (ANOVA). The least significant differences among the treatment means were determined at *p* ≤ 0.05 by using the Tukey test. Moreover, a second one-way analysis was carried out to investigate the storage time (in the supplements).

## 3. Results

### 3.1. Chemical Composition of Essential Oils and Free Radical Scavenging

The chemical composition of the EOs is shown in Table 1. A total of nine and 13 compounds were identified in the PMO and TTO, respectively. Levomenthol was reported to be the predominant compound (36.27%) in PMO followed by Cyclohexanone, 5-methyl-2-(1-methyl ethyl)-, Cyclohexanone, 5-methyl-2-(1-methyl ethyl)-, trans-, and D-Limonene. In TTO, 4-Terpinenol was reported to be the predominant compound (42.56%) followed by Gamma-Terpinene, Alpha-Terpinene and Terpineol.

In this study, the free-radical scavenging activity of PMO and TTO were assessed through the DPPH radical scavenging test. The radical scavenging activity was observed in EOs at the concentration of 50 µL mL^−1^ for each PMO and TTO. PMO had lower radical scavenging activity (54%) compared to TTO (63.56%).

### 3.2. Appearance

**Figure 1:**
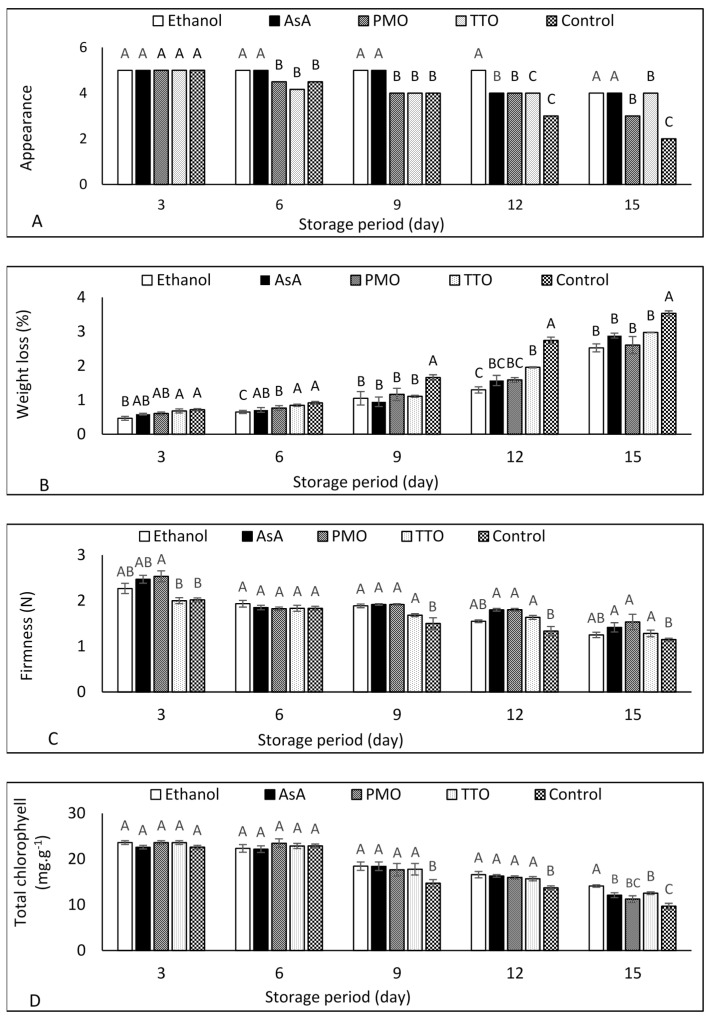
Effect of ethanol, ascorbic acid (AsA), peppermint oil (PMO), and tea tree oil (TTO) on (**A**) appearance, (**B**), weight loss, (**C**) firmness, and (**D**) total chlorophyll of fresh-cut green bean pods stored at 5 °C for 15 d. Different letters indicate significant differences (*p* < 0.05) using the Tukey test at every storage point. Data are means of three replicates. Vertical bars represent standard error.

The appearance score was gradually lowered during storage time (results presented are from one-way ANOVA analysis for each treatment compound, with storage time being the factor, Supplementary Table S1). At the end of storage (15 d), the control samples showed the lowest appearance score (Figure 1A), whereas the ethanol and AsA treatment showed the highest appearance score from 6 d storage until the end of the storage period in comparison to other treatments. For PMO and TTO, a gradual decrease in appearance was observed during the storage period.

### 3.3. Weight Loss

Weight loss in samples increased during the storage period for all treatments (Supplementary Table S1). The samples treated with ethanol showed lower levels of weight loss in comparison to control during the entire storage time, and the lowest on days 6 and 12 among treatments (Figure 1B). At the end of the storage, the control samples showed the highest level of weight loss. Moreover, AsA, TTO, and PMO treatments reduced the weight loss when compared to the control from 9 to 15 d.

### 3.4. Firmness

A reduction in firmness was observed for all the samples with increasing storage period (Supplementary Table S1). There was no difference in firmness between samples treated with ethanol and the control during storage periods except after 9 d of storage (Figure 1C). Moreover, treatments with AsA, PMO and TTO were equally effective in maintaining firmness at higher levels than controls from day 9 and thereafter. It was also shown that no difference was observed in firmness between treatments with TTO and PMO and the control only after three and six days of storage (Figure 1C).

### 3.5. Chlorophyll Content

Chlorophyll content decreased with an increase in storage time (Supplementary Table S1). These results indicated that there was no difference among treatments after three and six days of storage. However, all treated samples had higher chlorophyll content compared to the control after 9 d and 12 d, whereas ethanol resulted in the highest chlorophyll content after 15 d (Figure 1D). Samples treated with AsA, and TTO had higher chlorophyll content than the control 15 d of storage.

### 3.6. Vitamin C

**Figure 2:**
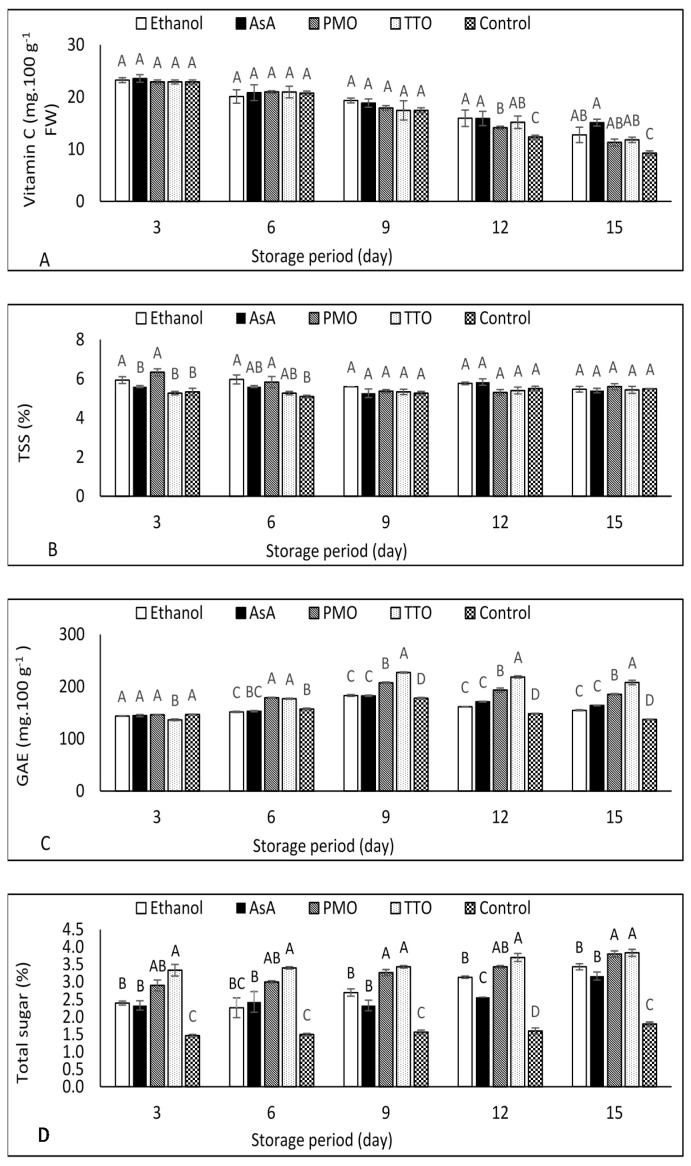
Effect of ethanol, ascorbic acid (AsA), peppermint oil (PMO), and tea tree oil (TTO) on (**A**) vitamin C, (**B**) total soluble solids (TSS), (**C**) total phenolic compound (TPC), and (**D**) total sugars of fresh-cut green bean pods stored at 5 °C for 15 d. Different letters indicate significant differences (*p* < 0.05) using the Tukey test at every storage point. Data are means of three replicates. Vertical bars represent standard error.

The vitamin C content of fresh green beans decreased with increasing storage time (Supplementary Table S1). The difference among treatments from 3 up to 9 d of storage was not noticeable, while all treatments showed higher vitamin C content compared to the control from 12 to 15 d of storage (Figure 2A).

### 3.7. Total Soluble Solids (TSS)

TSS of green beans samples decreased at the beginning of storage in all samples and then slightly increased in AsA, PMO and controls at the end of storage (Supplementary Table S2). The results in Figure 2B indicated that samples treated with ethanol and PMO had a higher TSS than the control after 3 d of storage. However, for the last three storage periods (9, 12, and 15 d), there was no major difference among all treatments.

### 3.8. Total Phenolic Compounds (TPC)

TPC increased up to 9 d of storage, then slightly decreased in all treatments and control (Supplementary Table S2). TPC was higher in all the treatments when compared to control from 9 d until the end of storage (Figure 2C). TTO treatment exhibited the highest TPC levels, followed by PMO from day 9 and afterwards.

### 3.9. Total Sugars

The total sugar content of samples treated with ethanol, PMO, TTP, and control slightly increased during the storage time, while the concentration in samples treated with AsA did not change substantially (Supplementary Table S2). As shown in Figure 2D, samples treated with either ethanol or AsA maintained a higher total sugar than the control, but lower than the remaining treatments from day 9 and thereafter.

Samples treated with either PMO or TTO exhibited a higher total sugar content compared to the control and other treatments without a significant difference between them (Figure 2D).

### 3.10. Mould, Yeast and Total Microbial Count

Mould and yeast (MY) and total microbial count increased with increasing storage time (Supplementary Table S2). In this study, the samples treated with ethanol suppressed the MY and total microbial count until 12 d of storage (Table 2). Our study showed that samples treated with either PMO or TTO suppressed MY and total microbial count during storage until 9 d of storage.

## 4. Discussion

### 4.1. Chemical Composition of Essential Oils and Free Radical Scavenging

The chemical composition of EOs agreed with Wu et al. [25], who stated that PMO mostly composed of monoterpenes and their derivatives. Vasile et al. [32] showed previously that the TTO contained both light monoterpenes and numerous sesquiterpenes, which are represented as the main component in 4-terpineol, followed by Alpha-Terpinene and Gamma-Terpinene. These were some of the important constituents of PMO and TTO in our results.

Our results indicated that PMO had higher radical scavenging activity than the results reported by others [25] who found that IC50 was recorded at 500 µL mL^−1^, which is a very high concentration compared to the concentration used in this study (50 µL mL^−1^). Variations in the antioxidant potential of EOs have been reported, primarily due to the presence of conjugated double bond compounds, which serve as hydrogen/electron donors [33]. PMO and TTO are also able to scavenge free radicals, which are harmful to the body because of their antioxidant function. TTO is also known for its therapeutic properties such as anti-inflammatory, antibacterial, and anticancer activity [34].

### 4.2. Appearance

**Figure 3:**
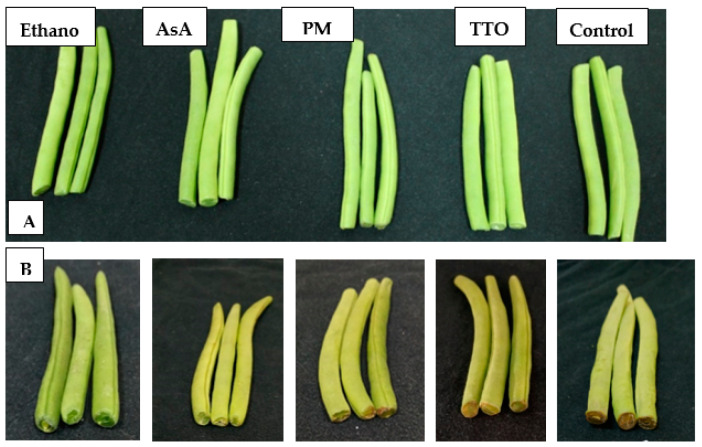
Green bean samples treated with ethanol, AsA, PMO, TTO, and control after (**A**) 3 d and (**B**) 15 d of storage at 5 °C.

Appearance is the most important quality parameter for the consumer’s acceptance. Figure 3 shows the appearance differences among treatments after 3 and 15 d from storage of green bean samples. It has been reported that the quality and acceptability of stored shredded carrots was maintained by exogenous AsA application [35], which agrees with our result (Figure 1A). This could be due to the inhibitory effects of AsA on chlorophyll degradation, ripening, and senescence. A sensory evaluation of the overall acceptability of the products, over time, and according to the proposed treatments, would be desirable. Our results shown in Figure 1A agree with a number of previous studies [8,36] that reported that TTO treatment maintained the overall acceptability and appearance of strawberry fruit stored at 20 °C for 3 d. Exogenous application of PMO has been shown to retard ripening, maintain appearance and suppress the decay of Mangosteen fruit [37], primarily due to the preservative effect of EOs as an antioxidant as its major components (Table 1). However, the synergistic or antagonistic influence of one compound in a small proportion of the mixture must be recognized [38].

### 4.3. Weight Loss

Weight loss of fresh fruit and vegetables during cold storage is affected by factors such as the structure of the cuticle [39], transpiration, and respiration [17]. Our results agree with Jin et al. [23], who reported a gradual decrease of water loss in sweet melon treated with vapour ethanol stored at 23 °C for 19 d. The reduced weight loss of the green bean samples by the 1% ethanol treatment could be attributed to the inhibitory effects on ethylene biosynthesis resulting in a decrease respiration process [22]. Previously, Ali et al. [40] reported a considerable decrease in the weight loss of litchi fruit treated by AsA compared to untreated samples. They suggested that AsA treatment reduced senescence and metabolic activity, which leads to a reduction of weight loss.

Regarding EOs treatments, our results shown in Figure 1B agree with previous works, where weight loss was decreased in strawberry fruit treated with vapour TTO stored at 20 °C for 3 d [36]. Results in Figure 1B support our hypothesis that PMO could reduce the weight loss of green bean samples. The exogenous application of PMO on dragon fruit reduced water loss during storage at 25 °C for 21 d [12]. Water loss reduction by the application of EOs is primarily due to EOs acting as a semi-permeable layer, resulting in a decrease in the respiratory rate, evaporation, and transpiration [4].

### 4.4. Firmness

The results in Figure 1C agree with the findings of other researchers [41] who reported that the firmness of Chinese bayberry is not affected by ethanol treatment. In previous studies, the ethanol-treated loquat fruit showed lower firmness values as compared to the non-treated fruit [18]. It has been reported that ethanol vapour treatment maintained the firmness of sweet melon during storage [23]. Therefore, more research is required to investigate the influence of ethanol treatment on the firmness of fresh fruit and vegetables.

AsA treatment retained the firmness of the green bean pods (Figure 1C); similar results were shown in previous works, where sweet pepper and plum fruit treated with AsA had higher firmness than the control due to its antioxidant properties [15,42]. The higher firmness of green bean samples treated with AsA could be explained by higher scavenging of ROS of cells leading to a decreased respiration rate [43]. It is well known that slowing oxidation preserves freshness and colour in fruits and vegetables.

EOs maintained the firmness of treated samples with either TTO or PMO could be due to its properties to inhibit the pectin degradation on the surface of green beans [44]. Additionally, EOs can act as a coating agent, which has a positive effect on respiration rate, leading to a reduction in loss of firmness [12].

### 4.5. Chlorophyll Content

It has been confirmed that ethanol retards chlorophyll degradation [45], repining and senescence [46]. The maintained chlorophyll content of the samples treated with ethanol is due to its inhibited effects on the activities and gene expression of chlorophyll enzymes [21]. It has been reported that AsA reduced the degradation of chlorophyll, which is correlated to photosystem and therefore resulted in higher chlorophyll content [47]. Our results shown in Figure 1d are in concurrence with Barzegar et al. [42], who found that AsA treatment maintained chlorophyll content in stored sweet pepper and green chilies, respectively.

Our results indicated that PMO and TTO treatments had a positive role in maintaining the chlorophyll content of fresh-cut green beans. This effect can be explained by a lower breakdown of chlorophyll pigments induced by PMO treatment, which acts as a coating agent [48]. Moreover, the thin coating layer around samples formed by TTO treatment could protect them from oxidation and colour changes [49].

### 4.6. Vitamin C

Vitamin C is classified as a natural antioxidant compound and has a potential role in reducing the risk of cancer by scavenging ROS in the human body [50]. However, vitamin C is rapidly degraded in fresh fruit and vegetables by several factors including storage [17]. Therefore, there is a need to minimize the decrease in vitamin C during refrigerated storage. Our results showed that the applied treatments of essential oil, ethanol and AsA presented a higher vitamin C value only towards the end of the storage period (9 and 12 d) The same results have been observed in previous studies for treated loquat fruit [18] with ethanol and litchi fruit with AsA [40].

The effect of postharvest treatment of TTO on vitamin C content of fruit and vegetable is not identified well due to limited research on this application. For example, a higher concentration of vitamin C was observed in lettuce plants treated before harvest with TTO and stored for 20 d at −2 °C [51]; however, it was mentioned that no significant difference was observed after 3 d of storage. The difference in our study could be due to the difference in time of application and different concentration of TTO, also due to the difference in plant spices.

Our results shown in Figure 2A are in concurrence with previous work by Naeem et al. [48], who found that application of PMO acting as a semi-permeable coating around the samples’ surfaces resulting in a reduction of vitamin C loss. The reduction in vitamin C loss by EOs application could be explained by the antioxidant properties of EOs resulting in a reduction in oxygen diffusion and respiration rate [52].

### 4.7. Total Soluble Solids (TSS)

Treatment with either ethanol or PMO resulted in a higher TSS in green bean samples compared to control (Figure 2B). Previously, Wang et al. [18] observed a higher TSS content in loquat fruit treated with ethanol compared with the control. Additionally, our results agree with early work reporting a gradual increase in TSS in mangosteen fruit treated [37] with PMO. Higher retention of TSS in samples treated with PMO can be related to the reduction of evaporation transpiration and respiration rate leading to conserving TSS [53].

### 4.8. Total Phenolic Compounds (TPC)

It is well known that TPC are considered the most important antioxidant compounds, responsible for scavenging free radicals, resulting in higher antioxidant defense [54]. Our results shown in Figure 2C agree with Wang et al. [18], who reported that TPC in loquat fruit increased at the beginning of storage and then decreased during the end of storage. In this study, ethanol and AsA treatments conserved the TPC of samples during storage. Additionally, it has been reported that that TPC in mung bean sprouts and litchi fruit was increased by AsA treatment, respectively [16,40]. Higher TPC in white bottom mushrooms treated with PMO compared to the control was recorded, consistent with the results seen in this study (Figure 2C) [55]. One of the explanations for higher TPC is that PMO have phenolic compounds that accumulate and result in higher TPC. Moreover, EOs also affect delaying the senescence process, resulting in maintaining TPC [37].

Samples treated with TTO showed higher TPC in this study; this agrees with previous work that observed higher TPC content during refrigerated storage of lettuce heads treated with TTO [51]. They also mentioned that this result could be ascribed to the induction of PAL by TTO, resulting in higher biosynthesis of phenolic compounds. Additionally, the potential of EOs antioxidant activity could reduce the oxidation of phenolic compound [56].

### 4.9. Total Sugars

In this study, either ethanol or AsA slowed the reduction of total sugar during cool storage (Figure 2D). Previously [57], a higher total sugar content in stored bitter melon was recorded when treated with ethanol plus melatonin. Another study indicated that treatment with AsA inhibited the reduction of the sugars content in strawberry fruit during cold storage [58]. This result could be due to the reduction of PAL activity by AsA treatment resulting in a lower loss of sugars during storage [59]. Total sugar preservation in samples treated with either PMO or TTO could be explained by the role of EOs in reducing the activity of enzymes that convert starch to sugar in stored crops [53]. Our results agree with previous work, where a reduction in total sugar loss was recorded in mushrooms treated after harvest with PMO compared to control [55].

### 4.10. Mould & Yeast (MY) and Total Microbial Count

Controlling postharvest diseases by ethanol application has been tested by various previous reports; e.g., *Botrytis cinerea* was controlled in table grape [60] and anthracnose in loquat fruit [18]. The reduced fungal growth in samples treated by ethanol is due to its striking lethal interactions with fungal spore that occurred in the mitochondrial membrane [61]. In this study and previous study on rocket [62], AsA treatment reduced MY and total microbial count. Regarding AsA application, it reduces the pH; a lower pH value is unsuitable for microbial growth [35].

Previous works demonstrated the inhibited role of EOs and their several compounds for controlling human and plant pathogens [4]. Our results in Table 2 show that both PMO and TTO had an inhibitory effect on microbial growth on the samples surface. The same trend was confirmed by previous work on dragon fruit [12], where PMO inhibited the mould growth during storage for 21 d. It was found that TTO treatment suppressed the growth of the pathogens on the surface of strawberry fruit during refrigerated storage [36], mostly due to inhibitory effects of the main chemical compositions of PMO such as Levomenthol [63] and TTO such as 4-Terpinenol [64] against microbial growth (Table 1).

## 5. Conclusions

Our study suggested that ethanol, AsA, PMO, and TTO can extend the life of fresh-cut green beans pods under refrigerated conditions by reducing weight loss and maintaining appearance, chlorophyll content, firmness, vitamin C, TSS, total sugar, and TPC. Moreover, a reduction in the mould and yeast count and total microbial count during storage was observed. Ethanol was the most effective application, followed by PMO and TTO, for preserving quality during refrigerated storage at 5 °C up to 15 d. Although all the treatments had positive effects, the best treatment shall be selected based on particular requirement and economic feasibility. For example, if there is a problem of higher microbial load along the value chain, we recommend the use of ethanol as it was the most effective, followed by PMP, TTO. Moreover, further research is required to enhance the storage ability of fresh-cut green beans and other legume crops including peas and board bean.

## Figures and Tables

**Table 1:** Identified volatile compounds of peppermint and tea tree essential oils.

Compound	Rt (min) *	(%)
**Peppermint oil (PMO)**		
Bicyclo [3.1.0]hex-2-ene, 4-methyl-1-(1-methylethyl)-	8.17	1.3
Bicyclo [3.1.0]hexane, 4-methylene-1-(1-methylethyl)-	9.592	1.97
D-Limonene	11.43	11.19
Decanal	11.53	2.41
Isopulegol	15.63	1.67
Cyclohexanone, 5-methyl-2-(1-methylethyl)-, cis-	15.92	25.32
Cyclohexanone, 5-methyl-2-(1-methylethyl)-, trans-	16.3	14.68
Levomenthol	16.6	36.27
Cyclohexanol, 5-methyl-2-(1-methylethyl)-, acetate, (1.alpha.,2.alpha.,5.beta.)-	20.84	5.19
**Tea tree oil (TTO)**		
Alpha.-Pinene, (-)-	8.16	5.71
(-)-.beta.-Pinene	9.58	2.55
Alpha.-Terpinene	11.00	10.77
Benzene, 1-methyl-3-(1-methylethyl)-	11.29	6.36
D-Limonene	11.43	3.24
Eucalyptol	11.50	2.06
Gamma.-Terpinene	12.53	10.12
Alpha.-Terpinolene	13.59	3.60
trans-β-Terpineol	15.60	0.49
4-Terpinenol	16.76	42.56
Terpineol	17.23	7.91
γ-Terpineol	17.48	1.43
Caryophyllene	24.90	3.19

* Retention time (RT) (as minutes).

**Table 2:** Effect of ethanol, ascorbic acid (AsA), peppermint oil (PMO), and tea tree oil (TTO) on mould and yeast and total count (log CFU/g) of fresh-cut green bean pods stored at 5 °C for 15 d. Data are mean of three replicates ± standard errors. Different letters indicate significant differences (Tukey test, *p* < 0.05%).

	Mould and Yeast (CFU/g)				
	3 d	6 d	9 d	12 d	15 d
Ethanol	ND *	ND	ND	ND	1.35 ± 0.03 c
AsA	ND	1.28 ± 0.12 b	1.37 ± 0.03 b	1.48 ± 0.09 ab	1.32 ± 0.02 c
PMO	ND	ND	ND	1.44 ± 0.03 b	1.49 ± 0.01 b
TTO	ND	ND	ND	1.41 ± 0.03 b	1.37 ± 0.02 c
Control	ND	1.54 ± 0.04 a	1.66 ± 0.01 a	1.68 ± 0.01 a	1.79 ± 0.03 a
	**Total count (CFU/g)**				
	**3 d**	**6 d**	**9 d**	**12 d**	**15 d**
Ethanol	ND	ND	ND	ND	0.53 ± 0.03 d
AsA	ND	ND	0.63 ± 0.06 b	0.90 ± 0.06 c	1.30 ± 0.05 c
PMO	ND	ND	ND	1.47 ± 0.01 b	1.49 ± 0.01 b
TTO	ND	ND	ND	1.55 ± 0.02 b	1.56 ± 0.04 b
Control	ND	1.71 ± 0.01 a	1.76 ± 0.03 a	1.93 ± 0.02 a	2.05 ± 0.03 a

* ND: not detected (there is no fungal growth found).

## Data Availability

Not applicable.

## Supplementary Materials

The following are available online at https://www.mdpi.com/article/10.3390/foods10051103/s1, Table S1: Effect of ethanol, ascorbic acid (AsA), peppermint oil (PMO), and tea tree oil (TTO) on appearance, weight loss, firmness, total chlorophyll, and vitamin C of fresh-cut green bean pods storied at 5 °C for 15 d, Table S2: Effect of ethanol, ascorbic acid (AsA), peppermint oil (PMO), and tea tree oil (TTO) on total soluble solids (TSS), and total phenolic compound (TPC), mold and yeast (CFU/g), and total count (CFU/g) of fresh-cut green bean pods storied at 5 °C for 15 d.
