# Supplementary material for: Extending the Shelf-Life of Fresh-Cut Green Bean Pods by Ethanol, Ascorbic Acid, and Essential Oils
Source: Foods. 2021 May 17;10(5):1103. doi: 10.3390/foods10051103 (PMC8156079; doi:10.3390/foods10051103)
Supplement: Supplementary file 1 [file foods-10-01103-s001.zip › foods-1210950-supplementary.pdf]

Supplementary table 1: Effect of ethanol, ascorbic acid (AsA), peppermint oil (PMO), and tea tree oil (TTO) on appearance, weight loss, firmness, total chlorophyll, and vitamin C of fresh-cut green bean pods storied at 5°C for 15 d. Different letters indicate significant differences between every storage point ($p < 0.05$) using Tukey test at the same column. Data are means of three replicates.

	Appearance				
	Ethanol	AsA	PMO	TTO	Control
3 d	5.00 a	5.00 a	5.00 a	5.00 a	5.00 a
6 d	5.00 a	5.00 a	4.50 ab	4.16 b	4.50 a
9 d	5.00 a	5.00 a	4.00 b	4.00 c	4.00 b
12 d	5.00 a	5.00 a	4.00 b	3.00 d	3.00 c
15 d	5.00 a	4.00 a	3.00 c	3.00 d	2.00 d
	Weight loss (%)				
	Ethanol	AsA	PMO	TTO	Control
3 d	0.46 d	0.58 a	0.60 c	0.68 d	0.61 e
6 d	1.16 c	0.81 b	0.76 c	0.84 c	0.71 d
9 d	1.25 b	0.95 b	1.56 b	0.90 c	1.65 c
12 d	1.29 b	1.57 c	1.58 b	1.95 b	2.74 b
15 d	2.52 a	2.88 d	2.60 a	2.97 a	3.53 a
	Firmness (N)				
	Ethanol	AsA	PMO	TTO	Control
3 d	2.26 a	2.47 a	2.53 a	2.00 a	2.02 a
6 d	1.93 b	1.85 b	1.82 b	1.83 ab	1.83 b
9 d	1.88 b	1.91 b	1.91 b	1.68 ab	1.50 b
12 d	1.55 c	1.80 b	1.80 b	1.63 bc	1.33 b
15 d	1.25 d	1.41 c	1.53 c	1.28 c	1.25 c
	Chlorophyll content (mg.g-1 FW)				
	Ethanol	AsA	PMO	TTO	Control
3 d	23.6 a	23.60 a	23.60 a	23.60 a	23.60 a
6 d	22.34 a	22.17 a	23.46 a	22.85 a	22.88 a
9 d	18.44 b	18.416 b	17.67 b	17.78 b	14.73 b
12 d	16.58 bc	16.32 c	15.98 c	15.68 c	13.72 b
15 d	14.10 c	12.11 d	11.26 d	12.54 d	9.78 c
	Vitamin C (mg.100 g-1 FW)				
	Ethanol	AsA	PMO	TTO	Control
3 d	23.23 a	23.56 a	22.90 a	22.90 a	22.90 a
6 d	20.10 ab	20.84 ab	20.97 a	20.96 ab	20.75 a
9 d	19.33 ab	18.84 b	17.92 b	17.43 bc	17.46 b
12 d	15.93 bc	15.89 c	14.13 c	15.16 c	12.34 c
15 d	12.75 c	15.10 c	11.30 d	11.79 d	9.25 d

Supplementary table 2: Effect of ethanol, ascorbic acid (AsA), peppermint oil (PMO), and tea tree oil (TTO) on total soluble solids (TSS), and total phenolic compound (TPC), mold and yeast (CFU/g), and total count (CFU/g) of fresh-cut green bean pods storied at 5°C for 15 d. Different letters indicate significant differences between every storage point ($p < 0.05$) using Tukey test at the same column. Data are means of three replicates.

	TSS (%)				
	Ethanol	AsA	PMO	TTO	Control
3 d	5.93 a	5.60 a	6.33 a	5.26 a	5.33 a
6 d	5.96 a	5.60 a	5.83 b	5.26 a	5.09 b
9 d	5.60 b	5.26 b	5.36 c	5.33 a	5.27 a
12 d	5.76 bc	5.83 a	5.30 c	5.40 b	5.50 a
15 d	5.46 c	5.40 b	5.60 b	5.43 b	5.48 a
	TPC (mg GAE. 100 g-1 FW)				
	Ethanol	AsA	PMO	TTO	Control
3 d	143.60 d	138.17 d	146.02 d	136.40 d	156.68 d
6 d	151.57 c	142.87 c	178.78c	176.77 c	187.27 b
9 d	163.04 b	156.49 b	207.36 a	227.01 a	204.86 a
12 d	171.43 a	182.51 a	193.38 b	218.52 ab	168.30 c
15 d	154.62 c	144.00 c	175.34 c	208.10 b	137.05 e
	Total sugars (%)				
	Ethanol	AsA	PMO	TTO	Control
3 d	2.53 b	2.33 a	2.90 c	3.33 c	1.46 b
6 d	2.26 b	2.43 a	3.00 bc	3.40 bc	1.50 ab
9 d	2.70 b	2.66 a	3.26 bc	3.43 bc	1.55 ab
12 d	3.46 a	2.90 a	3.43 ab	3.70 ab	1.60 ab
15 d	3.43 a	3.10 a	3.80 a	3.83 a	1.80 a
	Mold and yeast (CFU/g)				
	Ethanol	AsA	PMO	TTO	Control
3 d	0.00 b	0.00 b	0.00 b	0.00 b	0.00 b
6 d	0.00 b	1.28 a	0.00 b	0.00 b	1.54 a
9 d	0.00 b	1.37 a	0.00 b	0.00 b	1.67 a
12 d	0.00 b	1.48 a	1.44 a	1.41 a	1.69 a
15 d	1.35 a	1.32 a	1.49 a	1.37 a	1.79 a
	Total count (CFU/g)				
	Ethanol	AsA	PMO	TTO	Control
3 d	0.00 b	0.00 d	0.00 b	0.00 b	0.00 c
6 d	0.00 b	0.00 d	0.00 b	0.00 b	1.72 b
9 d	0.00 b	0.63 c	0.00 b	0.00 b	1.77 b
12 d	0.00 b	0.90 b	1.47 a	1.55 a	1.93 ab
15 d	0.53 a	1.30 a	1.49 a	1.56 a	2.05 a
